# Inflammatory choroidal neovascular membrane after healed tuberculous choroidal granuloma

**DOI:** 10.3205/oc000057

**Published:** 2017-03-03

**Authors:** Sikander A. K. Lodhi, Khadija Saifuddin, Santhosh Devulapally

**Affiliations:** 1 Sarojini Devi Eye Hospital / Osmania Medical College, Hyderabad. India

**Keywords:** tuberculosis, choroidal granuloma, exudative retinal detachment, CNVM, bevacizumab

## Abstract

**Objective:** To present a case of choroidal granuloma masquerading as intraocular tumor that healed on anti-tuberculous treatment but led to the development of inflammatory choroidal neovascular membrane (CNVM).

**Method:** A 42-year-old female patient with past history of hysterectomy presented with diminution of vision in the right eye. Fundus examination in the right eye showed a yellowish white choroidal mass with associated bullous retinal detachment superotemporal to fovea. Left eye fundus was normal. Fundus flourescein angiography showed early and late hyperflourescence with late pooling in serous detachments. Complete systemic evaluation did not yield a clue to diagnosis. Positron emission tomography scan (PET scan) showed enlarged lymph nodes in cervical, mediastinal and peritoneal regions. Lymph node biopsy showed caseating granulomas.

**Results:** The granuloma subsided and a scar formed 5 months after starting anti-tuberculous treatment with improvement in vision. Six months later, the vision deteriorated again with the development of a choroidal neovascular membrane (CNVM) at the margin of the scar. The CNVM resolved and all the signs of activity subsided after giving intravitreal antivascular endothelial growth factor (anti-VEGF) injections.

**Conclusions:** Making a diagnosis of tuberculous granuloma in a case of choroidal mass lesion is a challenge. PET scan helps in identifying metabolically active lymph nodes appropriate for biopsy. Healed scars of tuberculous choroid lesions should be followed closely to detect the development of CNVM.

## Introduction

Tuberculosis (TB) is a common infectious disease, nearly one-third of world population is infected with it. The estimated figure for prevalence of tuberculosis in India is given as 2.5 million [1]. It is estimated that 40% of the population in India is infected with tuberculous bacteria and that the majority of them has latent rather than active tuberculosis [2]. Tuberculosis primarily affects the lungs, but may also affect extrapulmonary organs including the eyes [3]. Tuberculosis can affect the anterior and posterior segments of the eye as well as the adnexa with various presentations. These pleomorphisms of lesions, as well as the lack of uniformity in diagnostic criteria, make a diagnosis of intraocular tuberculosis a challenge.

Choroidal neovascular membrane (CNVM) can arise as a complication of any pathologic process that involves retinal pigment epithelium and damages Bruch’s membrane [4]. The association between choroidal neovascular membrane (CNVM) and age-related macular degeneration or myopia is well known. CNVM may also develop as a complication in posterior uveitis; the reported incidence is 2% [5]. The different uveitic entities associated with CNVM include toxoplasmosis, punctuate inner choroidopathy (PIC) (17–40%), idiopathic multifocal choroiditis (MC) (33%), and serpiginous choroiditis [6]. It is also reported in ten eyes (9%) of seven patients in a follow-up of 58 patients (116 eyes) with Vogt-Koyanagi-Harada syndrome [7].

We report an interesting case of isolated choroidal granuloma without systemic tuberculosis in an immunocompetent young female that responded very well to anti-tuberculous treatment with good visual recovery. CNVM developed at the margin of the lesion that had regressed after tuberculostatic therapy. After four anti-VEGF injections, control of the CNVM was achieved. 

## Case description

**Figure 1:**
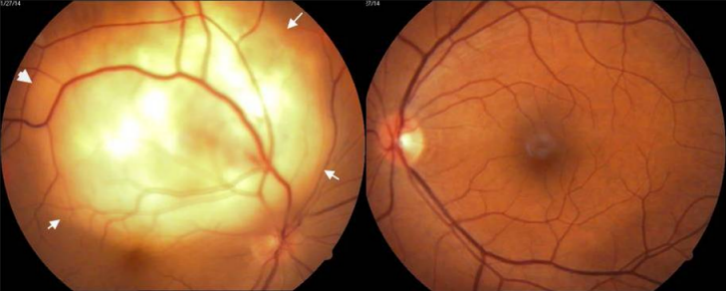
RE showing subretinal mass lesion. White arrows demarcating the outline of retinal detachment.

A 42-year-old female patient presented with a history of diminution of vision in the right eye for fifteen days. She had no other systemic complaints. She had undergone hysterectomy for an indication of dysfunctional uterine bleeding a year and a half ago. On examination, she had best corrected visual acuity of 20/400 in the right eye and 20/20 in the left eye. Both eyes anterior segment examination was within normal limits. Fundus examination of the right eye showed a yellowish white subretinal mass lesion six to eight disc diameters in size, located at the superotemporal arcade, just reaching the fovea with an overlying exudative detachment (Figure 1 (Fig. 1)). Left eye fundus was normal. The differential diagnosis included choroidal metastasis, choroidal tuberculoma or amelanotic melanoma. Because of hysterectomy in the recent past, the possibility of choroidal metastases was strongly considered. 

**Figure 2:**
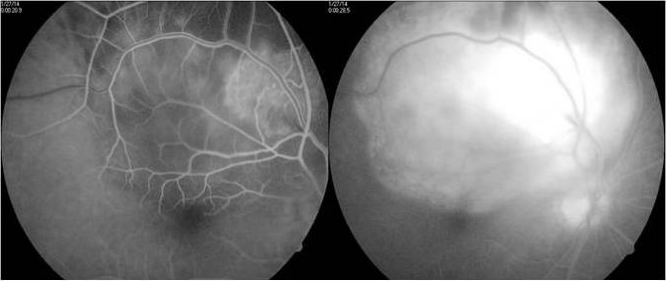
FFA shows early hyperflourescence with late pooling in serous detachment.

**Figure 3:**
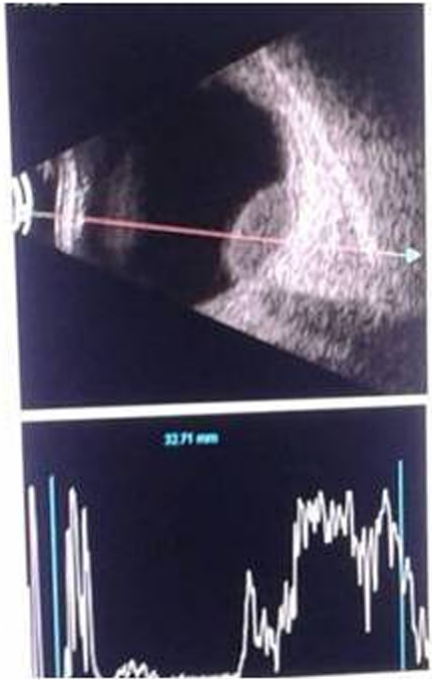
Ultrasonography shows a mass lesion of uniform echogenecity and low internal reflectivity.

Fundus flourescein angiography showed an early hyperflourescence in the region of the lesion with progressive increase in hyperflourescence and pooling of the dye outlining the serous detachment by late phases (Figure 2 (Fig. 2)). Ultrasonography showed a mass lesion of uniform echogenecity with low internal reflectivity (Figure 3 (Fig. 3)).

**Figure 4:**
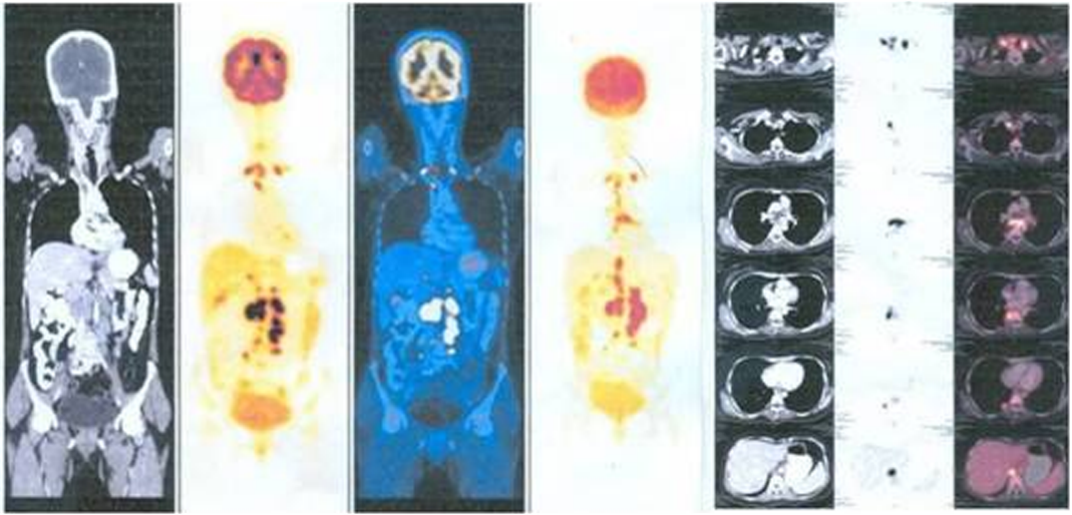
PET scan shows enlarged lymph nodes in cervical, mediastinal, and retroperitoneal regions.

**Figure 5:**
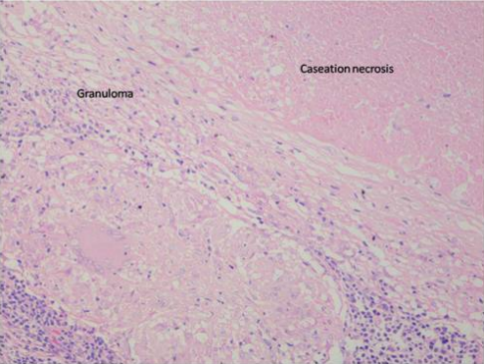
Showing granuloma with Langhan’s giant cells and caseation necrosis

The patient was referred to the physician for systemic evaluation. The patient underwent chest radiography, Mantoux test, complete blood picture, erythrocyte sedimentation rate (ESR) and HIV test. Positron emission tomography (PET) scan was advised by the physician. Chest x-ray was normal, Mantoux test (with 5 IU of purified protein derivatives) showed 15 mm induration. Erythrocyte sedimentation rate (ESR) was more than 20 mm. PET scan (Figure 4 (Fig. 4)) reporting was presence of multiple ^18^F-fluorodeoxyglucose (FDG) avid nodes (up to level IV) in cervical, mediastinal and retroperitoneal region – suggestive of an inflammatory pathology (tuberculosis) or metastases. Fine needle aspiration biopsy (FNAC) of cervical lymph node was inconclusive. Cervical lymph node biopsy showed caseous necrotic inflammation probably of tuberculous etiology. There was no evidence of neoplasia (Figure 5 (Fig. 5)). AFB culture of lymph nodes did not show any growth of acid fast bacilli.

**Figure 6:**
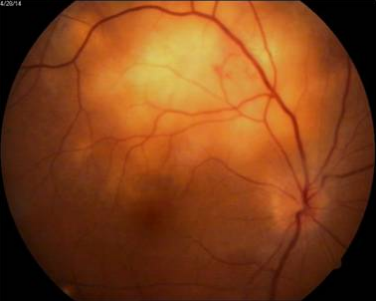
Healing lesion two months after starting anti-tuberculous treatment

**Figure 7:**
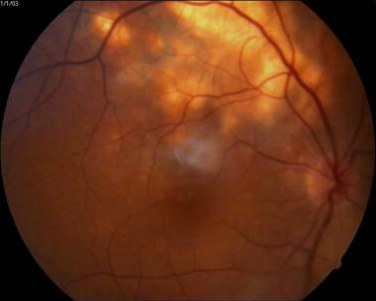
Scarred granuloma seven months after starting anti-tuberculous treatment. Fovea is spared.

The patient was started on four drug anti-tuberculosis treatment (isoniazid, rifampicin, etambutol, pyrazinamide with pyridoxine) with oral prednisolone (1 mg/kg body weight). Oral corticosteroids were tapered after the first four weeks and were stopped within eight weeks. The lesion started showing healing from the edges within two weeks after starting anti-tuberculous treatment (ATT) (Figure 6 (Fig. 6) and Figure 7 (Fig. 7)).

**Figure 8:**
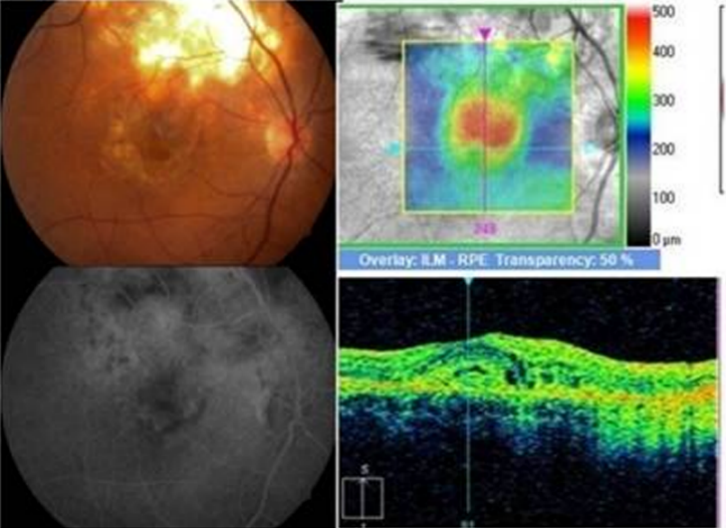
Juxtafoveal yellowish white lesion adjacent to healed scar with retinal edema, subretinal blood, exudates and subretinal fluid. FFA and SD-OCT showed signs of active choroidal neovascular membrane at the margin of the scar.

At ten-month follow-up, the scarred granuloma was stable with a visual acuity of 6/12p. A month later, still on ATT, the patient reported diminution of vision in the right eye, with best corrected visual acuity (BCVA) falling to 6/36p in RE. RE fundus examination showed macular edema with subretinal blood and exudates adjacent to the scar of the previous choroidal granuloma (Figure 8 (Fig. 8)). Fundus fluorescein angiography (FFA) showed patchy hypoflourescence due to subretinal blood and exudates and mild diffuse late leakage due to juxtafoveal CNVM. Spectral domain optical coherence tomography (SD-OCT) showed an increased foveal thickness, a dome-shaped hyperreflectivity at the level of the retinal pigment epithelium (RPE) and adjacent intraretinal cystic changes indicative of an active CNVM. 

**Figure 9:**
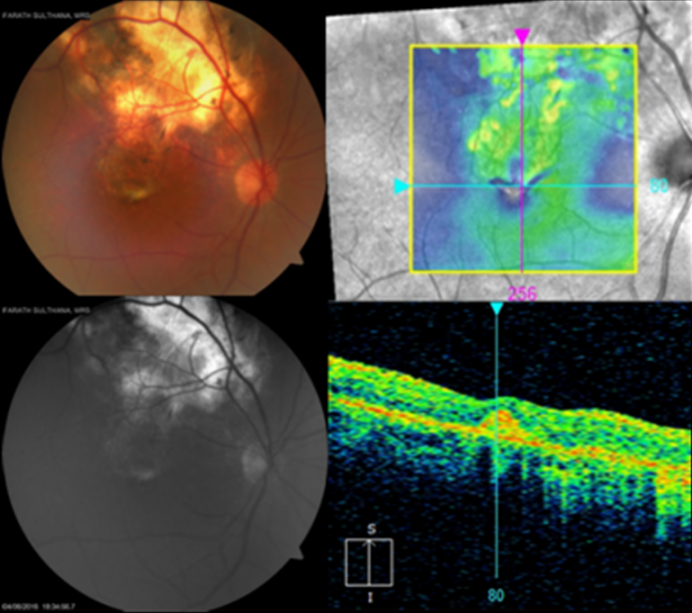
At one-year follow-up, fundus picture showed healed, pigmented scarred lesion at fovea. OCT showed reduced thickness, scarred lesion with partial restoration of foveal contour.

After informed consent, intravitreal anti-vascular endothelial growth factor (anti-VEGF) (Bevacizumab) was given. The CNVM resolved after four intravitreal Bevacizumab (Avastin) injections, leaving a scar with BCVA of 6/36 (Figure 9 (Fig. 9)).

## Discussion

Ocular tuberculosis may present with a variable clinical presentation including an intraocular mass. In addition, ocular manifestation is usually part of a systemic disease. Choroidal tuberculomata are result of a hematogenous spread of bacteria [8], [9]. Common clinical presentation of intraocular TB comprises of anterior uveitis (42%), posterior uveitis (36%), intermediate uveitis (11%), and panuveitis (11%). Tuberculous posterior uveitis may include choroidal tubercles, tuberculoma, serpiginous-like choroiditis, retinal vasculitis, sub retinal abscess, and endophthalmitis [10].

In this case, a history of hysterectomy with a subretinal mass lesion prompted a PET scan to look for any focus of malignancy. This identified metabolically active lymph nodes at several sites, including cervical nodes, which on biopsy identified caseating granulomas. This confirmatory histopathology allowed timely and appropriate treatment and presumably resulted in a better visual and anatomical outcome. PET/CT scans may suggest the sites of safe high-yield biopsies, thus yielding confirmatory histopathology, cultures, or PCR samples. Doycheva et al. found that ^18^F-FDG-PET/CT is useful in identifying metabolically active nodes appropriate for a biopsy that helps to establish the diagnosis and start appropriate treatment in presumed intraocular tuberculosis [11]. However, in the present case the development of choroidal neovascular membrane at fovea at the margin of the scar of regressed granuloma jeopardized the good visual outcome. CNVM, though uncommon, is a sight threatening complication of posterior uveitis. It results either from an inflammation induced angiogegenic drive or a degenerative disruption of RPE-choroid complex. The prime inducer of neovascularisation is the vascular endothelial growth factor [12]. The reported incidence of CNVM developing as a complication of uveitis is 2% [5]. Bansal et al. report 3.5% (5 eyes) developing CNVM in a study of 105 patients (141 eyes) of tubercular serpiginous like choroiditis with a minimum follow-up of 9 months [13]. Inflammatory CNVM develops close to the healed chorioretinal scar or choroidal granuloma – may be foveal, juxtafoveal and extrafoveal [14]. In this case, a juxtafoveal inflammatory CNVM has developed eleven months after the appearance of choroidal granuloma. Therapies available for inflammatory CNVM include photodynamic therapy (PDT), intravitreal bevacizumab and intravitreal ranibizumab [13]. In our case, the patient was continuing with ATT and the CNVM resolved and all the signs of activity subsided after four injections of intravitreal bevacizumab. The vision was stabilized at 6/36 and no recurrence occurred during a follow-up of one year. The median number of intravitreal anti-VEGF injections required to achieve an inactive CNVM in different studies is reported as 2 (range 1–5) [14]. In a study on “Risk of choroidal neovascularization among the uveitides” [5], Baxter et al. have concluded that CNVM is rare among cases of anterior and intermediate uveitis and found much more frequently (both at initial presentation as well at follow-up) in cases of posterior and panuveitis. CNVM is driven at least in part by the inflammatory process. Those cases that involve the region of the outer retina/retinal pigment epithelium (RPE)/choroid interface tend to have a higher risk of developing CNVM. 

## Conclusion

The present case of choroidal granuloma with diagnostic challenges had good visual recovery after ATT but the later development of CNVM has resulted in visual loss. Therefore, clinicians should educate the high-risk cases of posterior uveitis with potential symptoms of CNVM and provide them with screening tests like Amsler grid for an early detection and treatment. 

## Notes

### Competing interests

The authors declare that they have no competing interests.
